# Increased serum sTRAIL levels were correlated with survival in bevacizumab-treated metastatic colon cancer

**DOI:** 10.1186/1471-2407-12-58

**Published:** 2012-02-07

**Authors:** Atil Bisgin, Aysegul Kargi, Arzu D Yalcin, Cigdem Aydin, Deniz Ekinci, Burhan Savas, Salih Sanlioglu

**Affiliations:** 1 Human Gene and Cell Therapy Center of Akdeniz University Hospitals and Clinics, Department of Medical Genetics, Antalya, Turkey; 2 Department of Medical Oncology, Akdeniz University Faculty of Medicine, Antalya, Turkey; 3 Allergy and Clinical Immunology Unit, Antalya Education and Training Hospital, Antalya, Turkey

## Abstract

**Background:**

Colorectal cancer is the third most common cancer and the third leading cause of cancer-related death. Bevacizumab is a humanized monoclonal antibody developed against vascular endothelial growth factor (VEGF) for the treatment of metastatic cancer. The parameters of RECIST (Response Evaluation Criteria for Solid Tumors) are not adequate to detect important treatment effects and response. Our goal was to evaluate the possibility of using sTRAIL (serum-soluble TNF-related apoptosis-inducing ligand) and VEGF as markers of treatment efficacy and prognosis in patients with metastatic colon cancer.

**Methods:**

sTRAIL and VEGF levels were measured by ELISA in the sera of 16 bevacizumab-treated metastatic colon cancer patients and 10 presumably healthy age-matched controls. The measurements were taken before and after treatment for comparison purposes.

**Results:**

Elevated levels of sTRAIL were found in seven out of 16 patients after bevacizumab treatment. Although these patients had a median survival time of 20.6 months, the remaining bevacizumab-treated patients who did not show an increase in sTRAIL had a median survival time of 9.4 months. As expected, serum VEGF levels were decreased in all patients who received bevacizumab therapy and showed no correlation between serum VEGF levels and patient survival (data not shown).

**Conclusions:**

Serum sTRAIL levels might be a useful predictor of prognosis in metastatic colon cancer, in the early evaluation stages following bevacizumab treatment.

## Background

Colorectal cancer is the third most common cancer and the second leading cause of cancer-related death [1]. Overall, the 5-year survival rate is <10% for stage IV cancer [2]. The cure rate with surgery alone is very low and chemotherapy and radiotherapy are usually needed in patients with untreated metastatic colon cancer. The development of colorectal cancer is characterized by a sequence of events during which normal colonic epithelium gradually transforms to carcinoma tissue, in most cases, via the development of colorectal adenomas [3]. This sequence of events is driven by an accumulation of molecular (epi)genetic alterations causing progressive disorders in cell growth, differentiation and apoptosis [4,5]. Apoptosis, or programmed cell death, plays an important role in the development and maintenance of tissue homeostasis but also represents an effective mechanism by which abnormal cells, such as tumor cells, can be eliminated [6-8]. Abnormalities in apoptotic function or resistance to apoptosis have been identified as important events in the pathogenesis of colorectal cancer and its resistance to chemotherapeutic drugs and radiotherapy [9,10].

In recent years, bevacizumab, a novel humanized monoclonal antibody directed against vascular endothelial growth factor (VEGF) has found widespread clinical use as an angiogenesis inhibitor for certain types of metastatic cancers [11]. Treatment with bevacizumab with/without the combination of other chemotherapeutic agents inhibits VEGF receptor activation and vascular permeability, which eventually lead to tumor cell apoptosis [12,13]. Apoptosis can be induced passively, through the lack of essential survival signals, or actively, through the ligand-induced trimerization of specific death receptors of the tumor necrosis factor (TNF) receptor family, such as Fas, the TNF receptor, or TRAIL (TNF-related apoptosis-inducing ligand) receptor [14].

TRAIL (APO-2 ligand) is a transmembrane (type II) glycoprotein that also belongs to the TNF superfamily. The extracellular domain of TRAIL is homologous to that of other family members and shows a homotrimeric subunit structure. Like TNF and Fas ligand (FASL), TRAIL also exists physiologically in a biologically active soluble homotrimeric form, serum-soluble TRAIL; sTRAIL [15]. Several recent studies have indicated that sTRAIL is involved in the pathophysiology of different disease states such as cancer, viral infections, autoimmune diseases and inflammation, and defective apoptosis due to its interaction with its ligand preventing signaling for apoptosis may contribute to these diseases [16-21].

Several studies have shown that both the membrane-bound TRAIL and sTRAIL can induce apoptosis in a wide variety of tumor types by activating death receptors [22-24]. sTRAIL is also used as a positive marker for apoptosis [25]. However, it has been observed that the cytotoxic effects of antiangiogenic agents are increased in clinical phase II and III studies when these agents are combined with TRAIL-related therapies [26-28]. Another study with human glioblastoma cells has indicated that TRAIL inhibits angiogenesis stimulated by VEGF expression [29].

However, recently used RECIST parameters (Response Evaluation Criteria for Solid Tumors; responders vs. non-responders) are not adequate to document the differences in treatment response [30]. Therefore, there is a need for a sensitive, specific and reliable serum marker to monitor the therapeutic response.

The purpose of our study was to evaluate the possible use of sTRAIL as a marker for bevacizumab treatment efficacy at the cell apoptosis-linked step.

## Methods

The amount of released sTRAIL and VEGF was measured by ELISA in the serum of 16 metastatic colon cancer patients with liver metastases, who also received bevacizumab therapy, and 10 age- and sex-matched healthy control individuals who did not receive the treatment. Serum samples were obtained before bevacizumab-based chemotherapy regimens and 3 months after. Serum samples were kept between 2 and 8°C, centrifuged at 10,000 rpm for 10 min, and then frozen at -81°C until assayed.

ELISA was done using the Diaclone sTRAIL ELISA kit (Gen-Probe, Besancon, France) according to the manufacturer's instructions. Statistical analyses were performed using SPSS for Windows version 13.0 (SPSS Inc., Chicago, IL, USA). Group comparisons were made using the independent samples *t *test. Progression-free survival (PFS) was defined as the period from the beginning of chemotherapy until documented progression or death from any cause. Overall survival (OS) was defined as the period from the first day of treatment until the date of last follow-up or death.

Written informed consent relating to the Declaration of Helsinki was obtained from all patients. The study was approved by Akdeniz University Local Committee on Ethics.

## Results

**Figure 1:**
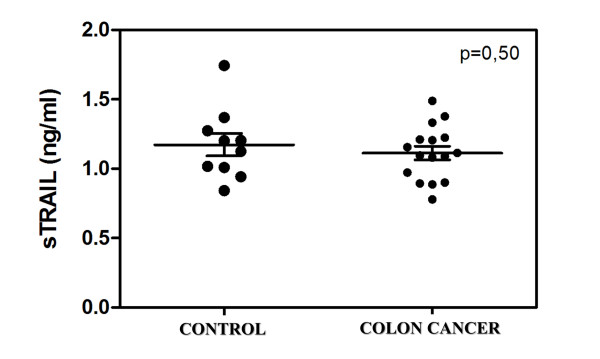
**Serum sTRAIL levels**. No difference between two groups (*P *= 0.50) before bevacizumab treatment: 1.12 ± 0.04 ng/ml in metastatic colon cancer patients and 1.17 ± 0.08 ng/ml in healthy control individuals.

**Figure 2:**
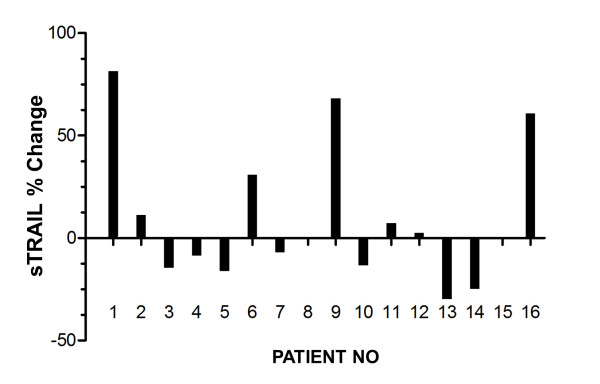
**Serum sTRAIL levels after treatment**. Elevated levels of sTRAIL were found in seven out of 16 metastatic colon cancer patients whose serum sTRAIL concentrations were similar to those of healthy age- and sex-matched control individuals (n = 10) before bevacizumab therapy, as seen in Figure 1. These increases were significant compared with pretreatment measurements (*P *< 0.001). The increase or decrease in the serum levels were shown as percentage change in serum sTRAIL.

The baseline clinical characteristics of the patients are summarized in Table 1. The serum sTRAIL concentrations before therapy were similar to those of the controls (Figure 1). Serum sTRAIL levels in metastatic colorectal cancer (MCRC) patients before bevacizumab-based chemotherapy and healthy controls were 1.12 ± 0.04 ng/ml and 1.17 ± 0.08 ng/ml, respectively. After bevacizumab treatment, seven out of 16 patients' post-treatment sTRAIL ratios were significantly increased as shown in Figure 2.

**Table 1:** Colon cancer patients versus healthy individuals.

		Healthy Controls (n = 10)	Colon Ca patients (n = 16)
Age		51, 8	55, 18

Sex	Male	8 (%80)	12(%75)
	
	Female	2(%20)	4(%25)

Age and sex distribution of metastatic colon cancer patients (n = 16) and healthy control individuals (n = 10)

Nine patients had progressive diseases, resulting in an overall response rate (56.2%). Median overall survival was 9.4 ± 0.9 months in non-responders. Interestingly, these nine patients were the same ones who showed no increase in sTRAIL levels after bevacizumab treatment. Therefore, we wanted to know whether there was any correlation between sTRAIL levels and overall survival rates. Our study demonstrated that elevated sTRAIL levels after bevacizumab treatment were significantly associated with increased median overall survival of up to 20.6 months (Table 2).

**Table 2:** Change in serum sTRAIL levels and association with survival.

*Response Status*	*Survival* *(month)*	*Serum sTRAIL Level (ng/ml)*
*Alive (n = 7)*	20,6 ± 0,5	1,38 ± 0,10
*Dead (n = 9)*	9,4 ± 0,9	0,92 ± 0,05

*Statistical analysis (unpaired* *t-test)*	*p *< 0,05 (*p *= 0,0001)	*p *< 0,05 (*p *= 0,0002)

Metastatic colon cancer patients who were treated with bevacizumab, and had increased levels of serum sTRAIL after treatment, had an extended median survival time up to 20.6 months. In contrast, median survival was 9.4 months for patients with decreased or unchanged sTRAIL after the bevacizumab therapy

Not surprisingly, the serum VEGF levels were decreased in all patients who received bevacizumab therapy (data not shown). Serum VEGF levels in MCRC patients before bevacizumab-based chemotherapy were 211 ± 189 ng/ml, and 3 months after treatment, they decreased to 117 ± 18.9 ng/ml, similar to healthy controls (119.5 ± 35.1 ng/ml).

## Discussion and conclusions

MCRC is mainly treated with combination of bevacizumab and irinotecan or oxaliplatin-based chemotherapy. The role of TRAIL and another apoptotic marker FAS/FASL systems in CRC patients treated with chemotherapeutic agents has been discussed previously [31-33]. The use of sTRAIL is a novel concept for which few data exist. We evaluated sTRAIL ratio as a marker of chemotherapeutic responsiveness in MCRC patients. This is believed to be the first study in which changes in levels of sTRAIL after bevacizumab treatment have been estimated in overall survival of MCRC patients.

In this study, we compare the levels of sTRAIL in responders versus non-responders to bevacizumab therapy. In responding patients, sTRAIL level increased, but the level decreased or was unchanged in non-responders. This significant change may be a result of the effect of bevacizumab on cancer cells via apoptosis. Another study has found that, although there was no significant difference between sTRAIL, soluble death receptor (sDR)4 and sDR5 levels in MCRC patients before and after treatment, significant correlations were observed between post-treatment sFASL and sDR4, post-treatment sFAS and sTRAIL, post-treatment sTRAIL and sFAS/sFASL ratio, and post-treatment sFASL and sDR5 [34]. This controversy may also have been due to the patients enrolled in the study. Although we chose newly diagnosed patients who had bevacizumab as first-line treatment, in the other study, patients were mostly treated with bevacizumab-based chemotherapy in the second-line setting after failure of oxaliplatin-based chemotherapy.

There have been many studies that have focused on identifying biomarkers for prediction of bevacizumab efficacy. The most obvious protein biomarker to select for testing bevacizumab efficacy is VEGF, but no studies have yet found changes in VEGF concentrations during therapy to have consistent predictive value [35]. One study has found that circulating levels of intercellular adhesion molecule (ICAM)-1 to be at least potentially predictive of survival benefit. Other studies have suggested that neutropenia might have a negative effect on bevacizumab efficacy. There have also been other studies that have concluded that polymorphisms in the VEGF pathway may have predictive value [36,37]. However, although several candidate biomarkers have already been described, including tumor and plasma VEGF, circulating E-selectin, ICAM-1 and vascular cell adhesion molecule-1, much more work is needed to determine the most effective biomarkers and thus to select the patients that are most likely to benefit from bevacizumab therapy [36].

As a conclusion, our study gave a different perspective on MCRC and bevacizumab treatment efficacy in relation to apoptosis and serum sTRAIL levels. The present data highlight the potential importance of sTRAIL in this setting. Further study with a larger patient population and longer duration is warranted to clarify its value.

## Competing interests

The authors declare that they have no competing interests.

## Authors' contributions

AB carried out the immunoassays, participated in the design and execution of the study and drafted the manuscript. AK followed up the patients and carried out the survival analysis. ADY performed the statistical analysis and participated in its coordination. CA assisted AB with immunoassays. DE carried out VEGF immunoassays and blood sampling. BS followed up the patients and participated in the study design. SS participated in study design and coordination. All authors read and approved the final manuscript.

## Acknowledgements

This study was supported by the Akdeniz University Scientific Research Project Administration Division and Health Science Institute.

## Pre-publication history

The pre-publication history for this paper can be accessed here:

http://www.biomedcentral.com/1471-2407/12/58/prepub
